# Supplementary material for: HIVprotI: an integrated web based platform for prediction and design of HIV proteins inhibitors
Source: J Cheminform. 2018 Mar 9;10:12. doi: 10.1186/s13321-018-0266-y (PMC5845081; doi:10.1186/s13321-018-0266-y)
Supplement: Supplementary file 2 — Additional file 2. Source code of HIVProtI web server. [file 13321_2018_266_MOESM2_ESM.zip › HIVprotI_Source-code/hivprotI/weka-3-6-6/doc/weka/gui/boundaryvisualizer/package-tree.html]

weka.gui.boundaryvisualizer Class Hierarchy


---


|  |  |  |
| --- | --- | --- |
| **Overview** \| **Package** \| Class \| **Tree** \| **Deprecated** \| **Index** \| **Help** \| **Weka's home** | |  |
| **PREV**   **NEXT** | **FRAMES**    **NO FRAMES**     **All Classes** |  |


---


## Hierarchy For Package weka.gui.boundaryvisualizer

**Package Hierarchies:**: All Packages

---

## Class Hierarchy

- java.lang.Object
  - java.awt.Component (implements java.awt.image.ImageObserver, java.awt.MenuContainer, java.io.Serializable)
    - java.awt.Container
      - javax.swing.JComponent (implements java.io.Serializable)
        - javax.swing.JPanel (implements javax.accessibility.Accessible)
          - weka.gui.boundaryvisualizer.**BoundaryPanel**
            - weka.gui.boundaryvisualizer.**BoundaryPanelDistributed**
            - weka.gui.boundaryvisualizer.**BoundaryVisualizer** (implements weka.core.TechnicalInformationHandler)
    - weka.gui.boundaryvisualizer.**KDDataGenerator** (implements weka.gui.boundaryvisualizer.DataGenerator, java.io.Serializable)- weka.gui.boundaryvisualizer.**Messages**- weka.gui.boundaryvisualizer.**RemoteBoundaryVisualizerSubTask** (implements weka.experiment.Task)- weka.gui.boundaryvisualizer.**RemoteResult** (implements java.io.Serializable)

## Interface Hierarchy

- weka.gui.boundaryvisualizer.**DataGenerator**

---


|  |  |  |
| --- | --- | --- |
| **Overview** \| **Package** \| Class \| **Tree** \| **Deprecated** \| **Index** \| **Help** \| **Weka's home** | |  |
| **PREV**   **NEXT** | **FRAMES**    **NO FRAMES**     **All Classes** |  |


---
